# Patient Understanding of Multiple Myeloma and Communication Experiences (PUMMaCE): Results From a UK Survey

**DOI:** 10.1002/jha2.70347

**Published:** 2026-07-23

**Authors:** Valerie Jenkins, Rachel Hunter, Ruth Habibi, Shirley May, John Jones, Theresa Street, Clare Evans, Anna Cowley, Barbara Collinson, Helena Harder

**Affiliations:** ^1^ Sussex Health Outcomes Research & Education in Cancer Brighton & Sussex Medical School University of Sussex Brighton UK; ^2^ Patient Representative East Sussex England; ^3^ Clinical and Experimental Medicine BSMS Brighton & Sussex Medical School Brighton UK; ^4^ East Sussex Healthcare NHS Trust London England; ^5^ Department of Haematology KCH London UK

**Keywords:** communication, multiple myeloma, patients’ views

## Abstract

**Introduction:**

Multiple myeloma (MM) is a complex haematological cancer. The terminology used can make discussions more challenging. We designed an online patient survey to explore this area.

**Methods:**

Questions with fixed and free text response options explored understanding of MM and phrases Health Care Professionals (HCPs) and family used that were helpful or not.

**Results:**

Data from 138 patients revealed that most were satisfied with the HCPs' communication. Many gave examples where information received impacted their understanding, for example, about prognosis, treatment management and support.

**Conclusion:**

These findings will inform the production of educational materials to improve communication between patients, their families and HCPs.

**Trial Registration:**

The authors have confirmed clinical trial registration is not needed for this submission.

## 1 Patient Understanding of Multiple Myeloma and Communication Experiences (PUMMaCE): Results From a UK Survey

Results from NHS England's National Cancer Patient Experience Survey showed that only 63% of patients living with multiple myeloma (MM) fully understood their diagnosis [1]. This finding is unsurprising, as the terminology used to describe MM and available treatment regimens, including numerous drug combinations can be challenging to explain and difficult for patients and their families to comprehend. Many cancer charities provide MM patient information materials on their websites. However, these are predominantly in written form and often perceived by patients as factual or (overly) complex and negligent of the emotional consequences of the diagnosis [2]. Moreover, it is unclear how current materials help patients’ and families’ understanding of the condition. A lack of sufficient generic and disease specific Health Care Professional (HCP) communication training can lead to patient dissatisfaction and sub‐optimal shared decision‐making. This is reflected in complaints from a Swedish cancer care region, where patients reported not receiving proper information, not being listened to and treated impersonally [3]. Also, a review of communication barriers in a clinical setting revealed inadequate consultation time, making it difficult for patients to engage with or process sometimes challenging information [4]. All discussions between HCPs and patients, especially those outlining complex issues present difficulties when the topic is a life‐threatening condition. While there are several generic HCP communication skills courses, there are fewer on how to manage the specific concerns affecting thousands of individuals living with MM.

We conducted a national online survey (PUMMaCE: Patient **U**nderstanding of **M**ultiple **M**yeloma **a**nd **C**ommunication **E**xperiences) to inform the production of future educational materials for patients, their families and Haematological HCPs. PUMMaCE was designed following a literature review [5], interviews with Haematologists, specialist nurses and patient representatives, to elicit examples of gaps in communication and/or information that was challenging to discuss or patients found particularly difficult to understand. Six short sections with fixed and free text response options allowed respondents to share thoughts about phrases used that helped or hindered their understanding of the diagnosis, treatments and management (Supporting Information). They also described some of the helpful and not so helpful behaviours exhibited by family and friends. PUMMaCE was advertised from January to April 2025, and our collaborators shared the survey link via social media, MM charities, support groups and word of mouth. PUMMaCE was supported by Brighton & Sussex Medical School (BSMS) and the UK Charity Comskills4health Ltd. (Charity number: 1143360) and approved by the BSMS Research and Governance Ethical Committee (Ref: ER VAL/1).

Standard descriptive statistical analyses were used to explore the data, and the results give a current view of the information gaps patients in the UK have about their diagnosis, prognosis, treatment management and support. Most respondents (*n* = 138:56 male, 81 female, 1 did not state gender) had been living with MM for either 1–3 years (42.7%; 59/138) or 4–10 years (33.3% 46/138). Over three quarters (107/138) were partnered, 47.8% (66/138) retired and 27.5% (38/138) had caring responsibilities (see Table 1 for other demographics). The cohort included patients treated intensively with autologous stem cell transplant, first line therapy, maintenance therapy and treatments for relapsed disease. Examples included ‘combination myeloma therapy’ (e.g., IRD, IPD, dara/Velcade), single agent oral maintenance, and injected single agent anti‐CD38 directed maintenance,' but few were in clinical trials.

**TABLE 1:** Characteristics of PUMMaCE survey respondents.

	*N* = 138
**Age Group (years)**	
31–40	6 (4.3%)
41–50	29 (21%)
51–60	38 (27.5%)
61–70	34 (24.6%)
71–80	28 (20.3%)
81–90	3 (2.2%)
**Ethnicity**	
White British	121 (87.7%)
White European	5 (3.6%)
White other	2 (1.4%)
Black British	3 (2.2%)
Afro‐Caribbean	1 (0.7%)
Black other	1 (0.7%)
Asian British	1 (0.7%)
Asian other	1 (0.7%)
Mixed ethnicity	2 (1.4%)
Prefer not to answer	2 (1.4%)
**UK Region**	
NE England	2 (1.4%)
NW England	6 (4.3%)
Yorkshire	6 (4.3%)
East Midlands	10 (7.2%)
West Midlands	10 (7.2%)
London	14 (10.1%)
SE England	39 (28.3%)
East England	8 (5.8%)
SW England	16 (11.6%)
NI	4 (2.9%)
Scotland	16 (11.6%)
Wales	1 (0.7%)
Channel Isles	5 (3.6%)
Prefer not to say	1 (0.7%)
**Highest level of education**	
No educational qualifications	1 (0.7%)
GCSEs (O levels)	20 (14.5%)
A Levels	11 (8%)
Vocational	12 (8.7%)
University	55 (39.9%)
Post grad	34 (24.6%)
Prefer not to answer	5 (3.6%)
**Employment status**	
Not in paid employment	6 (4.3%)
Retired	66 (47.8%)
Medically retired	8 (5.8%)
Long term sick	8 (5.8%)
Part time	15 (10.9%)
Full time	23 (16.7%)
Self employed	10 (7.2%)
Other	2 (1.4%)

Although haematologists (69.6%; 96/138) usually gave the diagnosis in person (87%; 120/138), many patients were meeting this HCP for the first time (73.3%; 88/120) and were often unaccompanied (35.8%; 43/120). In common with many haematological disorders, the general public have few reference points or prior familiarity of someone having MM, which resulted in 75% (104/138) indicating that they had never heard of MM before their diagnosis. Participants suggested that HCPs ought to include patients’ close partners/friends in treatment and management discussions, if needed, allowing them to gain a better knowledge of MM. This might result in carers feeling more equipped to provide practical and supportive behaviours. One way to achieve this is by offering recordings of the consultations, which patients could share with others at their discretion. Although this practice is routine in some oncology clinics only five people in PUMMaCE, reported receiving one. Recordings are an important tool for shared decision making, particularly when the information is complex; they are shown to improve patients’ knowledge and understanding without, importantly, increasing anxiety [6, 7, 8, 9, 10]. It offers an opportunity for the patient to listen again (alone or with others) in a less stressful context, for example, at home, and make notes of questions to ask their haematologist or specialist nurse.

Overall PUMMaCE respondents were satisfied with their treatment and management discussions (84.8%; 117/138), despite the complex terminology, and 80.4% (111/138) had access to specialist nurses unlike many patients with solid tumours, for example, metastatic breast cancer [11]. Participants’ understanding of MM was reflected in the language they used to describe the condition to their family and friends. They focused either on the nature of the disease, for example, *Blood cancer that affects the bone marrow*, or its clinical implications, for example, *Multiple myeloma causes me to have a lot of infections and causes a lot of fatigue*. Seven respondents, however, found the treatment aims difficult to understand, and despite no HCP being reported to have used the term ‘cure’, three nonetheless believed that this was the intended outcome.

Prognostic consultations were inherently challenging; only 46.6% (56/120) of participants had discussed it and three quarters (42/56) had to initiate the topic themselves. Some HCPs hold well‐intentioned but misguided notions that patients may lose hope if told, will ask if they wish to know, or that prognostic estimates are too uncertain to predict with accuracy, particularly given the rapidly evolving therapeutic landscape [12]. The improved treatment options for MM, specifically the targeted and immunotherapies have resulted in many people surviving longer. When one line of treatment stops working there are often other options available. Whilst positive, it can delay prognostic discussions and, in some cases, give false hope. A study of clinicians treating advanced disease patients (including MM), reported widely inconsistent estimations of life expectancy with and without treatment [13]. Even when clinicians believed that only 51% of patients would derive some medical benefit, active treatments were prescribed to those for whom benefit was unlikely or uncertain [13]. In an excellent series of studies, Kiely and coworkers [14, 15] suggest offering patients typical, best‐ and worst‐case scenarios based on median survival from clinical trials. In addition to being more accurate it gives patients a realistic expectation of what might happen and open up these often‐challenging discussions. Together, these studies highlight the challenges surrounding prognosis and treatment decision‐making and may help explain the low rate of clinician initiated prognostic conversations in our survey. There is a pressing need for further research into patients’ understanding of prognosis to help develop educational interventions to facilitate these discussions.

The PUMMaCE free text responses resulted in 248 comments about things HCPs said that helped or hindered patients’ understanding of MM, followed by general behaviours that were positive or less so. Table 2 illustrates some of these. Effective communication skills included the use of lay language, clear explanations, and allowing questions. In contrast, using complex terminology, not listening to patients’ concerns, a lack of facts and not answering questions directly hampered comprehension. HCPs were viewed in a positive light, providing support and clear information; however, there were some examples of insensitive comments and behaviours.

**TABLE 2:** Illustrative quotes about behaviours that helped and did not help.

	Helpful	Not helpful
What things did the HCPs say that helped or hindered understanding of your MM diagnosis?	*'Explaining the disease in simple terms and drawing diagrams; Answering emails quickly or phoning to answer queries.'* Female ID 171 *'Helped by being clear and honest.'* Male ID 184	*'Using multiple terms for things such as autograft for autologous stem cell transplant and plasma cell neoplasm for cancer cells was a bit confusing.'* Female ID 177 *'Out of date literature was given to me, suggesting my prognosis was 3mths to 3 years.'* Male ID *269*
What things did the HCPs, do or say that helped or did not help?	*‘Having the number of the support nurse. The best thing is referring me to the Late Effects clinic. I don't think I would have lasted this long without it’*. Female ID 161 *‘They have gone out of their way to provide tests and other services to allow me to continue with booked holidays and the like*’. Male ID 306	*‘Giving the prognosis was a big shock. I was on my own. Giving me bad news when treatment didn't work, when I was on my own’*. Female ID 292 *‘Sent me letters with information in that I had no context for e.g. your bone marrow is 70% myeloma, your light chains are 2300’*. Male ID 251
What did family or friends do or say that helped or did not help?	*'I had a wonderful group of friends who scooped us up in the first year when I was in hospital a fair amount. They cooked for my husband and children and took the boys to sports and after school activities. They also took them out for treats during the summer holidays when I was unable to'*. Female ID 188 *'Lifts to the hospital. Visits when I was an inpatient. Supporting my decisions, e.g., to retire early'*. Male ID 175	*'Gossiped; Wanted me to do more than I was able during treatment; Asked too many probing questions for nosiness; Not visited; Looked at with pity'*. Female ID 198 *'Looking up my condition and giving advice that I don't want'*. Male ID 179

Participants generated 320 comments regarding helpful and unhelpful communication or behaviours of family and friends (Table 2). The majority were positive and showed that most appreciated and valued practical help, empathy and friends and family being available. Less helpful behaviours included using insensitive language, becoming more distant or being overprotective and giving unsolicited advice. A thematic analysis manuscript of the rich and sometimes moving free text comments is being prepared and will form the basis of future educational materials.

As PUMMaCE was a time‐limited online survey and recruitment was mainly via Charities and MM support groups, there was a lack of diversity. To address this imbalance, we will conduct focus groups with people from ethnic minority and /or disadvantaged backgrounds to explore their thoughts on the PUMMaCE findings. This might highlight similarities and differences and further inform future resources for family and friends. Also, we intend to design and pilot a workshop for haematology team members to enhance their communication with patients living with MM and would hope it would be as useful as one recently evaluated for HCPs working in metastatic breast cancer [16].

Navigating the complexities and consequences of a diagnosis of MM is difficult for all concerned. Results from the PUMMaCE survey provided an opportunity to examine patients’ perspectives of living with MM. Treatments are constantly improving, providing more patients with a genuine prospect of living longer and there is a need to increase the quality of the extended survival through the provision of better information and support services.

## Author Contributions

**Valerie Jenkins**: conceptualisation, survey content, methodology, interpretation of data, writing – original draft, writing – review and editing. **Rachel Hunter**: methodology, editing manuscript. **John Jones**: survey content, interpretation of data, writing – review and editing. **Theresa Street**: survey content, interpretation of data, writing – review and editing. **Clare Evans**: survey content, interpretation of data, editing manuscript. **Ruth Habibi**: creating online survey, data curation, analysis, writing – review and editing. **Shirley May**: creating online survey, data curation, analysis, writing – review and editing. **Anna Cowley**: interpretation of data, writing – review and editing. **Helena Harder**: interpretation of data, writing – review and editing. **Barbara Collinson**: survey content, interpretation of data, writing – review and editing.

## Funding

The authors have nothing to report.

## Ethics Statement

Brighton and Sussex Medical School Research and Governance Ethical Committee (Ref: ER VAL/1) approved the study.

## Consent

Participants completed online consent before completing the survey.

## Conflicts of Interest

The authors declare no conflicts of interest.

## Data Availability

Once educational materials have been created, the data that support the findings of this study are available from the corresponding author upon reasonable request.
